# CD19 Chimeric Antigen Receptor-Exosome Targets CD19 Positive B-lineage Acute Lymphocytic Leukemia and Induces Cytotoxicity

**DOI:** 10.3390/cancers13061401

**Published:** 2021-03-19

**Authors:** Shabirul Haque, Sarah R. Vaiselbuh

**Affiliations:** 1 Feinstein Institute for Medical Research, Northwell Health, 350 Community Drive, Manhasset, NY 11030, USA; svaiselbuh1@pride.hofstra.edu; 2 Department of Pediatrics, Staten Island University Hospital, Northwell Health, 475 Seaview Ave, Staten Island, NY 10305, USA; 3 Monsey Health Center, 40 Robert Pitt Drive, Monsey, NY 10952, USA

**Keywords:** exosomes, CD19 CAR, B-cell acute lymphocytic leukemia, CAR-T cell therapy

## Abstract

**Simple Summary:**

Our research describes our designer exosomes express CD19 Chimeric Antigen Receptor (Exo-CD19 CAR). This novel Exo-CD19 CAR is cytotoxic for CD19-positive leukemia B-cells without interfering with cytotoxicity in CD19-negative cells. This innovation can be translated into broader clinical applications as CD19 CAR exosome-based nano-immunotherapy for B-cell leukemia instead of whole CD19 CAR T-cell immunotherapy.

**Abstract:**

CAR-T cell therapy is not without some clinical adverse effects, namely cytokine storms, due to a massive release of cytokines when CAR-T cells multiply in the body. Our goal was to develop exosomes expressing CD19 CAR to treat CD19-positive B-cell malignancies, instead of using whole CD19 CAR-T cells, thereby reducing the clinical risk of uncontrolled cytokine storms. Exosomes are extracellular nanovesicles (30–150 nm), composed of lipids, proteins, and nucleic acids, that carry the fingerprint of their parent cells. Exosomes are a preferred delivery system in nano-immunotherapy. Here, HEK293T parent cells were transduced with CD19 CAR plasmids and cellular CD19 CAR expression was confirmed. Exosomes (Exo-CD19 CAR) were isolated from the conditioned medium of non-transduced (WT) and CD19 CAR plasmid transduced HEK293T cells. Consequently, CD19 B-lineage leukemia cell lines were co-cultured with Exo-CD19 CAR and cell death was measured. Our data show that Exo-CD19 CAR treatment induced cytotoxicity and elevated pro-apoptotic genes in CD19-positive leukemia B-cells without inducing cell death in CD19-negative cells. Overall, the novel CD19 CAR exosomes target the CD19 surface antigens of leukemic B-cells and can induce contact-dependent cytotoxicity.

## 1. Introduction

The CD19 surface antigen plays a critical role in B-cell development and maturation [1,2], and elevated levels of CD19 can lead to B-cell malignancies such as acute lymphoblastic leukemia (ALL), chronic lymphocytic leukemia (CLL), and B-cell lymphomas [3]. Expression of CD19 is not limited to cancer biology, but the expression of CD19 also plays a major role in the development of immunodeficiency disorders and autoimmune disorders such as rheumatoid arthritis and multiple sclerosis [4]. ALL is one of the most common hematological cancers in children but prevalent in the adult population as well [5,6]. Since the CAR T-cell therapy concept was first developed in pediatric ALL [7,8], there are already two FDA-approved CAR-T-cell immunotherapeutics on the market, namely tisagenlecleucel (Kymriah^TM^) for pediatric B-cell leukemia and axicabtagene ciloleucel (Yescarta^TM^) for the treatment of adult patients with relapsed or refractory large B-cell lymphoma. Both molecules are based on CD19-directed genetically modified autologous T-cell immunotherapy [9]. CD19 CAR T-cells have been tested in clinical trials with remission rates as high as 90% for relapsed/refractory B-ALL [10]. CD19 CAR T-cells trigger cell toxicity in CD19-positive B-cell leukemia and appears non-toxic to CD19-negative B-cells [11,12,13,14]. Clinical trial data demonstrated that CD19 CAR-specific monoclonal antibodies targeting and killing CD19-positive B-cell malignancies [15]. Unfortunately, both Kymriah^r^ and Yescarta^r^ can have severe side effects such as macrophage activation syndrome (MAS) and/or cytokine release syndrome (CRS), neurologic toxicities, tumor lysis syndrome (TLS), anaphylaxis, on-target, off-tumor toxicity, and B-cell aplasia after treatment [8,16,17]. Due to the high level of adverse effects from whole-cell based CD19 CAR-T cell immunotherapy, research communities are exploring the use of less reactogenic nano-biomolecules to lessen CAR immunotherapy side effects [18]. 

In brief, exosomes are lipid bilayer nanovesicles (30–150 nm in diameter) which belong to a sub-group of extracellular vesicles (EVs). Exosomes are secreted by almost all biological fluids of the body and composed of proteins, lipids, and coding/non-coding RNA [19,20]. Exosomal cargo reflects the nature of the parent cell and as such they can be loaded with a variety of favorable or potential cytotoxic molecules. Literature review educates that there is a growing interest in exosomes and their role in cancer [21]. In our previous work, we showed that silencing of exosomal miR-181a reverses pediatric acute lymphocytic leukemia cell proliferation [22]. Human cytotoxic T-lymphocyte (CTL)-derived exosomes contain surface membrane molecules (CD3z, CD8, and the TCRs), which can recognize and target tumor cells [23]. CTL-derived exosomes are loaded with harmful molecules such as granzymes, lysosomal enzymes, and perforin which may induce cytotoxic effect in target cells [24,25]. Human natural killer (NK)-cell-derived exosomes play a role in exosome-mediated cytotoxicity against solid tumors. In addition, CAR-T cells-derived exosomes that express antibodies that can recognize EGFR (epidermal growth factor receptor) and HER2/CD340 showed promising anti-tumor activity in breast cancer [26]. 

The novelty of our approach is that exosomes derived from any parent cells in vitro with CAR plasmids of choice to develop “off-the-shelf” targeted CAR exosomes. One of the major benefits of this approach is to cut short the cumbersome and time-sensitive step of culturing in vitro autologous T-cells to engineer CAR-T cells for subsequent infusion into the patient [7,8]. However, exosomes equipped with CD19 CAR molecules are only at a starting point and leave room for further exploration. To our knowledge, no other studies on exosomes engineered to express CD19-CAR that can target CD19-positive B-cell leukemia have been described. 

Briefly, we have demonstrated that exosomes (Exo-CD19 CAR) carry and display CD19 CAR molecules on their surface. Exo-CD19 CAR treatment induces cytotoxicity and pro-apoptotic gene augmentation in CD19-positive leukemia B-cells without inducing cell death in CD19-negative cells. Our findings support an innovative application of exosomes with potential for clinical translation, namely the use of CD19 Chimeric Antigen Receptor (CAR) exosomes as a novel approach for nano-immunotherapy in cancer. 

## 2. Results

### 2.1. Transfection of CD19 CAR Plasmids into HEK293T Producer Cells 

#### 2.1.1. Characterization of CD19 CAR Plasmid 

CD19 CAR plasmid was characterized and confirmed before using it in transfection experiments. Details of the plasmid are based on the vector map (Supplemental Figure S1A). The size of the plasmid was confirmed by agarose gel electrophoresis (Supplemental Figure S1B); and expression of eGFP, CD8a, and CD28 as inserts was confirmed by PCR on agarose gel (Supplemental Figure S1C).

#### 2.1.2. Transfection of Producer Cell Line 

**Figure 1:**
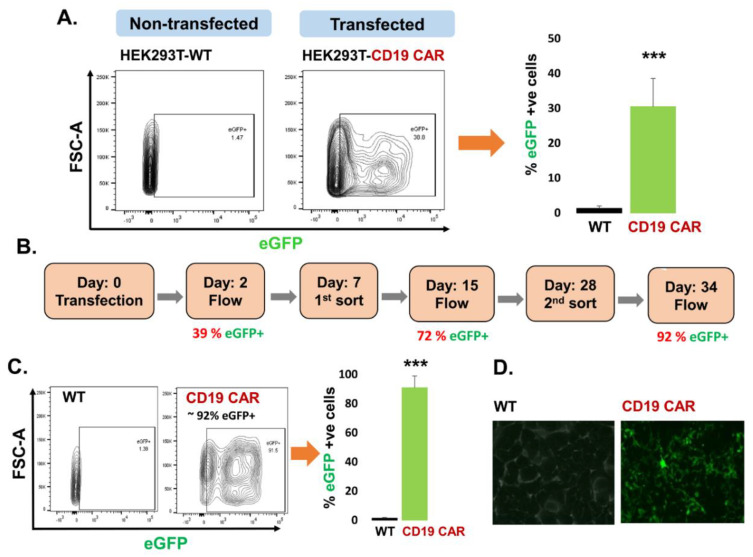
Transfection of the HEK293T producer cell line. (**A**) CD19 CAR plasmid transfection into the HEK293T cells based on eGFP expression (39%) compared to control non-transfected (WT) HEK293T cells by flow cytometry, (*n* = 3 independent experiments). (**B**) Timeline for enrichment of HEK292T cells transfected with CD19 CAR plasmid demonstrate stable transfection in enriched cells for more than a month. (**C**) Contour plot of flow cytometry confirming around 92% eGFP positive cells after 2nd round of cell sorting CD19 CAR positive HEK293T cells compared to non-transfected (WT) HEK293T cells, (*n* = 3 replicates). (**D**) Fluorescence microscopy demonstrating expression of eGFP in CD19 CAR transfected HEK293T cells. *p*-value (*** *p* < 0.001).

HEK293T cells were chosen as parent/host cells to enable production of CD19 CAR exosomes after transfection. CD19 CAR plasmid (eGFP tagged) was successfully transfected into the HEK293T cells and CD19 CAR expression was determined by the expression of eGFP by flow cytometry followed by cumulative data plotted as a bar graph (Figure 1A). The left panel shows non-transfected cells (HEK293T-WT) while the right panel demonstrates CD19 CAR transfected cells (HEK293T-CD19 CAR) with ~39% transfection rate. Consequently, HEK293T-CD19 CAR cells were sorted on low pressure to maintain cell viability in order to enrich the transfected cell population and allowing them to expand in cell culture after sorting. At day 0, transfection was carried out, on day 2, transfection was analyzed and if successful, cells continued to be cultured for 7 days for expansion. On day 7, the first round of cell sorting for enrichment was carried out and sorted cells were cultured and expanded for another week. On day 15, transfection was analyzed again by flow cytometer. On day 28, a second round of cell sorting for enrichment was carried out. At day 34, transfection was analyzed by flow cytometry as demonstrated (Figure 1B). After two rounds of cell sorting, ~92% eGFP positively transfected HEK293T cells were achieved as demonstrated by contour plot of flow cytometry and cumulative bar graph (Figure 1C). This data confirms that CD19 CAR plasmids are stably transfected in HEK293T cells for more than a month and can actively express a protein of interest. The expression of eGFP in HEK293T-CD19 CAR (transfected) cells was confirmed under fluorescent microscopy (Figure 1D). 

### 2.2. Confirmation of CD19 CAR Plasmid into the Transfected Producer Cell Line

**Figure 2:**
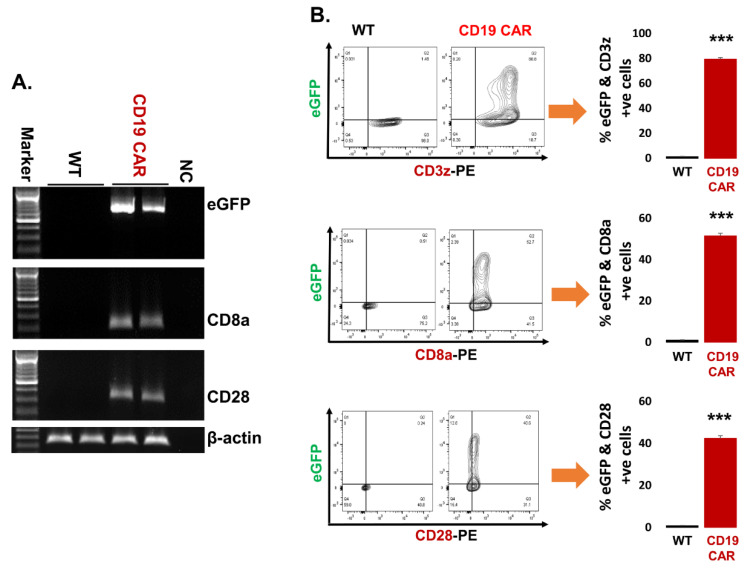
Confirmation of CD19 CAR plasmid into the transfected producer cell line. (**A**) Agarose gel demonstrating cellular mRNA expression (eGFP, CD8a, and CD28) in CD19 CAR plasmid transfected HEK293T cells and control non-transfected cells (WT-control). The β-actin band is same for each gene of interest because same sample/cDNA was used for PCR amplification of eGFP, CD8a, and CD28. (**B**) Protein expression by flow cytometry (contour plot) demonstrating surface protein expression of CD3z, CD8a, and CD28 co-localized with eGFP expression in CD19 CAR plasmid transfected HEK293T cells (CD19 CAR) and non-transfected cells (WT-control). Bar graph is representation of the three replicates. *p*-value (*** *p* < 0.001). This is a representation of cellular CD3z, CD8a, and CD28 expression.

In order to produce CD19 CAR exosomes, we first had to establish the success of CAR plasmid transfection into the parent cell line. The HEK293T-WT cells do not express eGFP and CD19 CAR components (CD3z, CD8a, and CD28). We confirmed the expression of CD19 CAR plasmid markers at both mRNA and protein levels in transfected parent cells (HEK293T-CD19 CAR). PCR-generated data shows that CD19 CAR transfected cells express eGFP, CD8a, and CD28 while non-transfected cells (WT) remained negative as controls (Figure 2A). The full image of gels are demonstrated (Supplemental Figure S6). Further, we also checked the expression of CD19 CAR components (CD3z, CD8a, and CD28) at the protein level. Both CD19 CAR transfected (CD19 CAR) and non-transfected (WT) cells were stained with respective antibodies and analyzed by flow cytometry demonstrated as contour plot and cumulative data represented as a bar graph (Figure 2B). Our results confirmed that there is expression of CD3z, CD8a, and CD28 along with eGFP in CD19 CAR transfected cells while there is no expression of the above molecules in non-transfected (HEK293T-WT) control cells.

### 2.3. Characterization of Purified Exosomes

**Figure 3:**
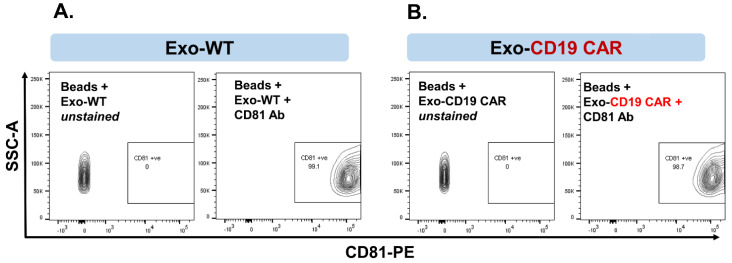
Characterization of purified exosomes. (**A**) Confirmation of CD63 and CD81 expression on exosomederived from the conditioned medium of HEK293T-WT by flow cytometry (contour plot). (**B**) Confirmation of CD63 and CD81 expression on exosomes derived from the conditioned medium of HEK293T-CD19 CAR by flow cytometry (contour plot).

After exosomes were harvested from culture medium (CM) of the HEK293T producer cells, they were characterized for authentication. We first established exosomal expression of CD63 and CD81 in the isolated nanoparticle fraction as signature biomarkers for exosomes. Both CD63 and CD81 are traditional exosomal markers and exosomes must express these markers [27]. Exosomes derived from CM of HEK293T-WT (non-transfected) cells are represented as Exo-WT. Exosomes derived from CM of HEK293T-CD19 CAR transfected cells are represented as Exo-CD19 CAR. Our data show that more than 99% of isolated and purified exosome particles are expressing CD81 and CD63 together in Exo-WT (Figure 3A) and Exo-CD19 CAR (Figure 3B). These results confirm that isolated exosomes are pure with good quality. 

### 2.4. Confirmation of CD19 CAR Molecules Expression in Harvested Exosomes

**Figure 4:**
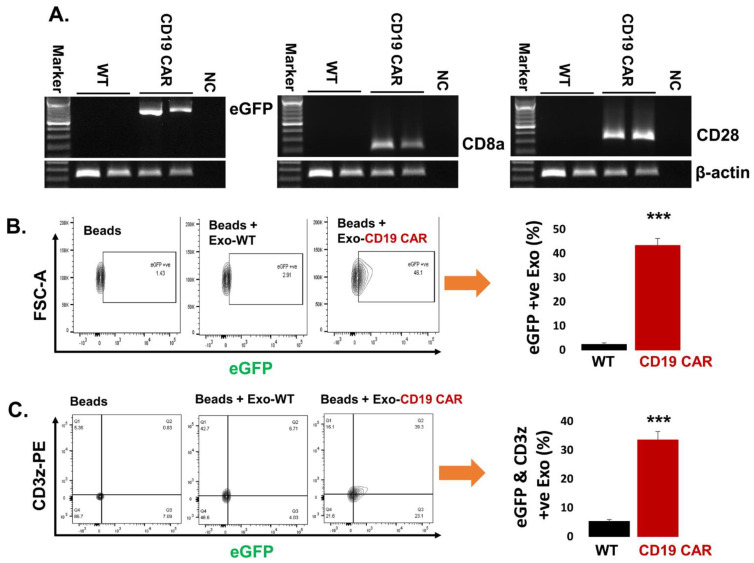
Confirmation of CD19 CAR molecules expression in harvested exosomes. (**A**) Agarose electrophoresis gel demonstrating Exo-CD19 CAR are positive for eGFP, CD8a, and CD28 mRNA expression while Exo-WT are negative for each of the markers. The β-actin band is same for each gene of interest because same sample/cDNA was used for PCR amplification of eGFP, CD8a, and CD28. (**B**) Flow cytometry contour plot demonstrating Exo-CD19 CAR expressing eGFP (unstained) compared with Exo-WT (control), and bar graph demonstrating cumulative data (*n* = 3 independent experiments). Exo-WT and Exo-CD19 CAR were coated with CD63 antibody capture beads. (**C**) Flow cytometry showing co-expression of eGFP with CD3z in Exo-CD19 CAR compared to Exo-WT (control), and bar graph demonstrating cumulative data (three replicates). Exo-WT and Exo-CD19 CAR were attached with CD63 antibody capture beads followed by staining with CD3z antibody. *P-value* (*** *p* < 0.001).

We wanted to make sure that the CD19 CAR molecules were present as exosomal cargo in exosomes harvested from HEK293T producer cell lines. Exosomal CD19 CAR molecular marker expression was analyzed by mRNA transcripts and protein expression. Exo-CD19 CAR showed positive CD19 CAR expression of mRNA transcripts (CD8a, CD28, and eGFP) while control Exo-WT demonstrated negative expression by PCR followed by agarose gel electrophoresis (Figure 4A). The full image of gels are demonstrated (Supplemental Figure S7). In addition, eGFP expression within Exo-CD19 CAR was confirmed by flow cytometry as demonstrated by contour plot and cumulative data represented by bar graph (Figure 4B) while Exo-WT appeared negative for eGFP expression. In addition, we also confirmed the expression of CD3z with eGFP co-localization. Exo-WT and Exo-CD19 CAR were coated with CD63 antibody capture beads followed by staining with a CD3z fluorescent antibody and analyzed by a flow cytometer as demonstrated by contour plot and cumulative data represented by a bar graph (Figure 4C). This evidence confirms that the Exo-CD19 CAR carry necessary components of the CD19 CAR molecular constructs as predicted, since exosomes can carry the cargo of their parent cells, in this case, the plasmids from the transfected HEK293T CD19 CAR producer cells. 

### 2.5. Delivery of Exo-CD19 CAR Molecules into the Target Cells

**Figure 5:**
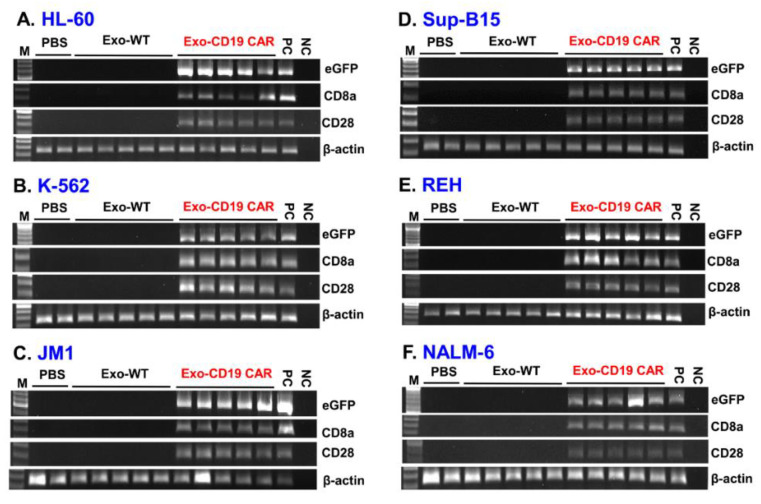
Delivery of Exo-CD19 CAR molecules into the target cells. Agarose gel shows eGFP, CD8a, and CD28 mRNA expression (CAR plasmid markers) in each of the target cells lines. (**A**) HL-60, (**B**) K-562, (**C**) JM1, (**D**) Sup-B15, (**E**) REH, (**F**) NALM-6. The β-actin is used as an endogenous control. Five different batches of exosomes (Exo-WT and Exo-CD19 CAR) were used in the experiments (*n* = 5). Densitometry of the gels are included as a supplementary figure (Supplemental Figure S3).

**Figure 6:**
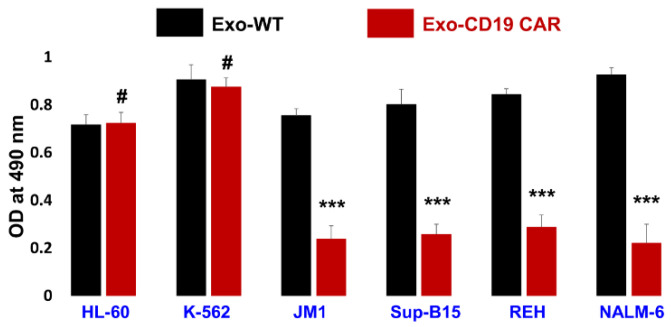
Cytotoxicity measurement by MTS assay. Exposure of Exo-WT and Exo-CD19 CAR on CD19-negative and CD19-positive cells. Exo-CD19 CAR exposure induced cytotoxicity in CD19-positive target cells (JM1, Sup-B15, REH, and NALM-6) while no cytotoxicity was observed in CD19-negative target cells (HL-60 and K-562). Combined data (*n* = 3 independent experiments) demonstrating level of significance and reproducibility. *P-value* *** *p* < 0.001 and # demonstrate not significant (*p* > 0.05). Black bar demonstrates Exo-WT treatment and red bar demonstrates Exo-CD19 CAR treatment.

We asked if Exo-CD19 CAR could target CD19-positive cells to deliver their exosomal cargo. Exosomes (Exo-WT and Exo-CD19 CAR) were co-cultured with several target cells: HL-60, K-562 (CD19-negative control cells), JM1, Sup-B15, REH, and NALM-6 (CD19-positive leukemic B-cell lines) at a concentration of 2.5 µg/µL. After 48 h of incubation, exposed target cells were harvested and cellular expression of plasmid eGFP, CD8a, and CD28 was determined by PCR. Expression of each marker was demonstrated in each of the target cell lines, HL-60 (Figure 5A), K-562 (Figure 5B), JM1 (Figure 5C), Sup-B15 (Figure 5D), REH (Figure 5E), NALM-6 (Figure 5F). Densitometry plot of all the gels is demonstrated including statistical analysis by image J (Supplemental Figure S3). The full image of each gel is provided as a supplemental figure: for Figure 5A HL-60 (Supplemental Figure S8), for Figure 5B K-562 (Supplemental Figure S9), for Figure 5C JM1 (Supplemental Figure S10), for Figure 5D Sup-B15 (Supplemental Figure S11), for Figure 5E REH (Supplemental Figure S12), for Figure 5F NALM-6 (Supplemental Figure S13). The β-actin was used as an endogenous control. Overall data confirm the delivery of Exo-CD19 CAR components (eGFP, CD8a, and CD28 mRNA transcripts) into each target cell compared to Exo-WT/PBS-treated control cells. Five different batches of purified Exo-WT and Exo-CD19 CAR were used in these experiments. It is important to note that HL-60 and K-562 cells also showed uptake of Exo-CD19 CAR components, while these cell lines do not express CD19. However, it is only in CD19-positive cells that Exo-CD19 CAR can induce cytotoxicity (as demonstrated Figure 6). Further, dose optimization of exosomes and confirmation of exosomal delivery into the target cells was carried out in Sup-B15 leukemic cells. Three different doses, 2.5, 5.0, and 7.5 µg/µL of exosomes (Exo-WT and Exo-CD19 CAR), were added to the target cells and co-cultured for 48 h. Cells were stained with eGFP antibody and expression of eGFP was analyzed by flow cytometry as demonstrated (Supplemental Figure S2A,B). It was observed that the optimal dose of exosomes was 2.5 µg/µL, since at higher doses, the delivery of exosomal contents reached a plateau. Therefore, we continued all future experiments with a working concentration at 2.5 µg/µL exosome load.

### 2.6. Exo-CD19 CAR Effect on CD19-Negative and CD19-Positive Cells 

To test our hypothesis whether Exo-CD19 CAR was able to target and have a cytotoxic effect on CD19-positive leukemia B-cells or not, we chose two types of target cells, namely CD19-negative control cells (HL-60 and K-562) and CD19-positive target leukemia cells (JM1, Sup-B15, REH, NALM-6). After four days of co-culture of Exo-WT and Exo CD19 CAR with above cell lines, cells were harvested and cytotoxicity was evaluated by MTS assay. Here, cumulative data are demonstrated as a bar graph (Figure 6). Our data support that Exo-CD19 CAR induces cytotoxicity in CD19-positive cells while having no cytotoxic effect in CD19-negative cells. This cytotoxicity was measured in comparison with Exo-WT (control). Interestingly, our data demonstrated that Exo-CD19 CAR treatment could not induce cytotoxicity in CD19-negative cells such as HL-60 and K-562, which showed no change in cell count, while cytotoxicity was robust in CD19-positive leukemic B-cells JM1, Sup-B15, REH, and NALM-6 with a reduction in cell counts. This data suggests that Exo-CD19 CAR discriminate between CD19 positive and CD19 negative cells leading to selective cytotoxicity based on CD19-negative and -positive expression status. The data show that Exo-CD19 CAR exposure induced cell death in CD19-positive leukemia B-cells while Exo-CD19 CAR exposure did not induce cytotoxicity in CD19-negative cells.

Further, we also demonstrated our data by flow cytometry (live cell numbers) in dot plots (Supplemental Figure S5A, left panel) and cumulative data from three different experiments were computed and demonstrated by bar graph (Supplemental Figure S5B, right panel). 

### 2.7. Exo-CD19 CAR Exposure Induces Pro-Apoptotic Genes in CD19-Positive B-Cell Leukemia

**Figure 7:**
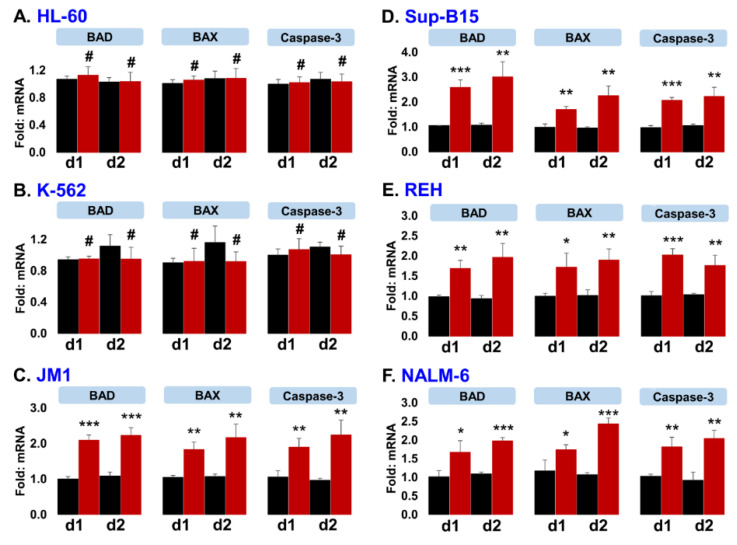
Exo-CD19 CAR exposure induces pro-apoptotic genes in CD19-positive B-cell leukemia. (**A**,**B**) The qPCR data show that Exo-CD19 CAR exposure could not induce pro-apoptotic genes (BAD, BAX, and Caspase-3) in CD19-negative cell lines (HL-60 and K-562). (**C**–**F**) The qPCR data demonstrate that Exo-CD19 CAR treatment induces pro-apoptotic genes (BAD, BAX, and Caspase-3) in CD19-positive cell lines (JM1, Sup-B15, REH, NALM-6). For quality and reproducibility purposes, five different batches of exosomes were utilized in the experiments (*n* = 5). Statistical *p*-value (* *p* < 0.05, ** *p* < 0.01, *** *p* < 0.001), # represent not significant. Red bars demonstrate Exo-CD19 CAR exposure, and black bars demonstrate control Exo-WT. d1 represent day 1 and d2 represent day 2 exposure of target cells with Exo-WT/Exo-CD19 CAR.

In order to identify the Exo-CD19 CAR cytotoxic effect at a molecular level, we explored pro-apoptotic gene expression in affected leukemic cells. Exo-WT and Exo-CD19 CAR exosomes were co-cultured with both CD19-negative and CD19-positive target cells. After 24 and 48 h of co-culture, cells were analyzed for mRNA expression of pro-apoptotic genes BAD, BAX, and caspase-3 by q-PCR. Interestingly, Exo-CD19 CAR exposure did not induce pro-apoptotic genes in CD19-negative cells HL-60 (Figure 7A) and K-562 (Figure 7B), while Exo-CD19 CAR exposure significantly induced pro-apoptotic genes in CD19-positive cells JM1 (Figure 7C), Sup-B15 (Figure 7D), REH (Figure 7E), NALM-6 (Figure 7F). These data confirm that Exo-CD19 CAR protein may induce cell death selectively in CD19-positive cells only.

Further, we measured cytokines IFN-g, TNF-a, and IL-2 mRNA expression by q-PCR in Exo-WT and Exo-CD19-CAR-treated cells. Cumulative data are included (Supplemental Figure S4). Data demonstrate that TNF-α mRNA and IL-2 mRNA are undetected in each cell line while very poor expression of IFN-g mRNA was detected in HL-60, Sup-B15, and K-562. The expression of IFN-g mRNA did not show significant difference in the Exo-CD19-CAR-treated group compared to Exo-WT (control) group.

## 3. Discussion

Cell-based immunotherapy is emerging as an interesting new approach as a treatment for hematological malignancies but is not without its own challenges. Especially, chimeric antigen receptor T-cell (CAR T-cell) immunotherapy is one of the most advanced options available at present. The cytotoxic effect and duration of CAR T-cell depends on the longevity of the engineered CAR T-cells in circulation. CAR T-cell replication or division also contributes to the strength of the immunotherapy in vivo. Pioneering work [7,28,29] described successful clinical application of CAR T-cell therapy as a rescue in children with second relapse acute lymphocytic leukemia. Soon after, the literature boomed with other CAR-concept clinical trials and their use in adult oncology solid tumors as well. There are many studies, aiming at different CAR-T constructs such as EGFR-specific CAR T-cells for non-small-cell lung cancer [30], CD19 CAR T-cells for leukemia/CD19-positive malignancies [12,13,15,31,32,33,34], and CAR T-cells with anti-programmed cell death protein 1 (PD-1) single-chain variable fragment (scfv) [35,36], all showing promising cytotoxic effect in cancer immunotherapy. In 2017, the Food and Drug administration approved the first two CAR-cell therapies for clinical use. Yescarta^r^ (B-cell lymphoma) and Kymriah^r^ (pediatric B-ALL) both are based on CAR T-cells that recognize CD19 on the surface of malignant B-cells [7,9]. Although these products provided good clinical outcomes with 90% remission rates in Phase I/II trial in refractory B-ALL, and overall survival of 78%, there is need for caution. Unfortunately, infused engineered CAR T-cells may provoke a cytokine storm in about 46% of patients due to massive cytokine release induced by T-cell proliferation. Cytokine release syndrome (CRS) results in a system inflammatory response syndrome (SIRS) with high mortality. Future development of CAR T-cell-based immunotherapy should be modulated to reduce this significant toxicity [37]. In our study, we propose a novel concept by introducing CD19 CAR expression into exosomes as carrier-vehicles of CD19 CAR protein components. Exosomes are small nanoparticles that are non-immunogenic since they are derived from the body’s parental cells and therefore are recognized as “self”. This adapted CAR-immunotherapy might reduce the risk of cytokine storms in patients [38]. 

HEK293T cells were transduced with CD19 CAR plasmids, and exosomes (Exo-CD19 CAR) were isolated from CM of HEK293T-CD19 CAR cells. Exosomes carry and display CD19 CAR molecules on their surface. RNA isolated from Exo-CD19 CAR showed evidence of exosomal mRNA expression of CD8a and CD28. Co-culture of target cells with Exo-CD19 CAR showed an induced cytotoxicity and pro-apoptotic gene induction in CD19-positive leukemia B-cells without any sign of cytotoxicity in CD19-negative cells. Interesting to note is that while CD19-negative control cells HL60 and K562 did show uptake of the Exo-CD19 CAR, this uptake did not result in any cytotoxic effect in these cells [21]. This is not surprising as it is known that exosomes are shed in the extracellular environment allowing for re-uptake by other cells by “fusion” (the membrane of the exosome merges with the target membrane) to deliver their cargo. Since exosomal miRNA (exo-miRNA) can be transferred to target cells and demonstrated functional activity, they have appropriately been called “exosomal shuttle RNA” (esRNA). Based on our observations, it appears that only when exosomal cell-entry is mediated via binding to the CD19 antigen on the surface of CD19-positive B-cells, it can open the gateway to downstream signaling that results in selective cytotoxicity.

Depending on the nature of the parent cell types and conditions, exosomes carry many ligands/proteins that are advantageous for immune cell proliferation and can be used as anti-tumor medicine. Dendritic cell-derived exosomes have demonstrated anti-tumor activity via promotion of natural killer (NK) cell proliferation and activation, since exosomes carry NKG2D ligand protein [39]. Potential killing mechanisms induced by NK-derived EVs/exosomes are cell death (DNA damage), apoptosis (caspase-3 and 7), necrosis (DNA fragment), and necroptosis (elF2a and caspase-12). The exosomal protein levels of PFN, GzmA, GzmB, and GNLY are deciding factors for the tumor lysis ability of NK-EVs. The packaging and orientation of these proteins into NK-EVs during EV biogenesis are a critical factor for cytotoxic strength, which can be exploited as a killing mechanism to target cancer cells [24]. All these are very promising anti-tumor molecules that can be potentially utilized for exosome-based nano-immunotherapy in cancer. Unfortunately, these anti-tumor molecules are non-specific and do not have the ability to target any specific malignant cells. Targeted specificity with minimal toxicity is very important and a critical feature to improve immunotherapy for translation into clinical use. 

There is some evidence that provides mechanistic insights in the functionality of exosome-CAR-based nano-immunotherapy. CAR-exosomes derived from effector CAR T-cells with cetuximab single-chain variable fragment (scfv) and trastuzumab scfv showed potent anti-tumor activity with minimal toxicity. Exosomes expressing cetuximab scfv recognize and kill EGFR-positive cells while exosomes expressing trastuzumab scfv recognize and kill HER-2 positive cells in breast cancer [26]. 

CAR exosomes as nano-immunotherapy have several advantages. First, the production of the cell-free exosomes is safer than live CAR T-cells; a case report showed that the CAR gene was accidentally introduced into a single leukemic B-cell during a CAR T-cell manufacturing process, as a result of which a patient developed resistance to CTL019 and ultimately died of progressive leukemia [40]. Second, exosomes have a nano-meter diameter size, which is a great advantage for the treatment of solid tumors, as nano-size therapeutic biomolecules can easily penetrate and access the tumor cells by biogenesis while large T-cells will have limited access to direct tumor tissue entry [21,41]. Third, exosomes expressing CAR utilized for nano-immunotherapy can overcome immunosuppressive side effects of the treatment [26].

There are many unanswered questions and concerns which need to be addressed because of the heterogeneity of exosomes. One such study compared the cytotoxic effect of CAR T-cells vs. CAR T-cell derived exosomes (which are exosomes harvested directly from engineered CAR T-cells), with data showing that 5 × 10^4^ CAR cells or 10 μg CAR exosomes could induce around 20% killing of 5000 tumor cells. The exact number of exosomes present in 1 μg of protein is controversial [42]. CAR expression on exosomes derived from CAR cells and CAR T-cells were quantified by ELISA, which showed around 0.25–0.69 ng CAR protein per 1 μg CAR T-cells based on the protein concentration, which corresponds to 10 ng CAR protein per 5 × 10^4^ CAR cells, while there are approximately 0.6 ng CAR protein per 1 μg CAR exosome [26]. We believe exosomes expressing CD19-CAR-based treatment have the following major advantages compared to existing CAR T-cell immunotherapy: 1. production of the cell-free exosomes is safer and easier than culturing CAR T-cells in vitro and allows for an “off-the-shelf’ production (exosomes are stable and can be frozen and thawed). 2. Exosome infusion into the patient is non-immunogenic and therefore will less likely induce a cytokine storm compared to whole CAR T-cells. 

## 4. Materials and Methods 

### 4.1. Cell Lines 

HEK293T cells were purchased as the parental producer cell line. HL-60 and K-562 were used as negative control cells, while JM1 and Sup-B15 as acute lymphocytic leukemia B-cell lines; and REH and NALM-6 as relapsed leukemic B-cells. Cell lines were purchased commercially by American Tissue Culture Cell bank (ATCC), HEK293T (CRL-3216, ATCC), HL-60 (CCL-240™, ATCC), K-562 (CCL-243™, ATCC), JM1 (CRL-10423™, ATCC), Sup-B15 (CRL-1929™, ATCC), REH (ACC 22, DSMZ), NALM-6 (CRL-3273™, ATCC). Each cell line was cultured and expanded as per manufacturer instructions. 

### 4.2. CD19 CAR Plasmid Isolation 

We selected a CAR-CD19 plasmid that was commercially available and had eGFP as a marker to first transfect the producer HEK293T cell line.

#### 4.2.1. Bacterial (*Escherichia coli*) Culture and Plasmid Isolation 

We obtained CAR-plasmid pHR CD19 CAR commercially that contains eGFP (as marker molecule) and CD3z, CD8a, and CD28 as CAR markers (Addgene, cat # 113015) [43]. Plasmids arrived in *E. coli* host cells stored in glycerol stock. *E. coli* host cells were expanded in liquid LB media with recommended antibiotics and incubated overnight. The next day, plasmid DNA was extracted by use of a DNA-extraction kit (A1223, Promega); DNA concentration and quality was verified by agarose gel electrophoresis. 

#### 4.2.2. CD19 CAR Plasmid Verification by Semi-Quantitative PCR 

Expression of eGFP, CD8a, CD28, and β-actin in the CAR-plasmid DNA was amplified by semi-quantitative PCR using the primers listed (Table 1). Agarose gel (1.5%) was prepared in 1x Tris/Borate/EDTA buffer with SYBR safe DNA gel stain (P/N S33102, Invitrogen). PCR products containing CAR plasmid DNA were mixed with 6x loading dye (R0611, Crystalgen) and loaded on the gel. After 25 min of electrophoresis at 200 V, the gel was exposed and the image was captured (Bio-Rad gel documentation system). 

### 4.3. Production of CAR-Exosomes in HEK293T Parent Cells

HEK293T cells were used as host cells to be transfected with the CAR-plasmid and as producer cells of CAR-exosomes as parent cells.

#### 4.3.1. Plasmid Transfection, Enrichment of Transfected Cells by FACS Cell Sorting, and Fluorescence Microscopy 

HEK293T cells (80–90% confluent in 100 mm tissue culture plate) were transfected with CD19 CAR plasmid (2.0 µg) by Effectene transfection reagent kit (301425, Qiagen) as per manufacturer’s protocol. Transfection efficiency (based on eGFP expression) was analyzed by flow cytometry after 48 h of incubation. Transfected HEK293T cells were sorted (BD FACSAria SORP) at low pressure. Two rounds of cell sorting were carried out to enrich to ~94% eGFP-positive HEK293T-CD19 CAR cells. In addition, eGFP-positive cells were imaged through fluorescence microscopy. 

#### 4.3.2. Cell Staining to Analyze Transfection Efficiency by Flow Cytometry 

To analyze transfection efficiency, we used the CAR-plasmid markers eGFP, CD3z, CD8, and CD28 and stained for flow cytometry as follows. HEK293T cells (1.0 × 10^6^), either non-transfected (HEK293T-WT) or transfected (CD19 CAR-plasmid), were washed with ice-cold PBS and fixed with 3% paraformaldehyde. Cell permeabilization was carried out with cold methanol (−20 °C) incubated for 30 min at −20 °C [44,45]. Cells were washed twice with washing buffer/staining buffer (5% sterile FBS in PBS + 0.1% sodium azide + 0.5 mM EDTA) and then re-suspended in 100 µL of staining buffer. Cells were stained with color-conjugated antibodies such as CD3z-PE-conjugated mAb (2-2479-82, eBioscience™), CD28-PE-conjugated mAb (12-0289-42, eBioscience™), CD8a-PE-conjugated mAb (12-0088-80, eBioscience™), GFP-PE conjugated antibody, (IC4240P, R&D). Each antibody was used at dilution 1:20 and incubated for 20 min in a dark cold room. Cells were washed twice with washing buffer (1–3 mL). The cell pellet was re-suspended in ice-cold PBS (150 µL) and fixed in 150 µL of 2% paraformaldehyde. Samples were read by flow cytometer (Fortessa).

### 4.4. Exosomes (Exo-WT and Exo-CD19 CAR) Production and Isolation

After confirmed enrichment of transfected HEK293T producer cells, we expanded the stable transfected cell line further to allow for exosome production and harvesting.

#### 4.4.1. Exosomes Production 

HEK293T-WT and HEK293-CD19 CAR-transduced cells were seeded at 1.0 × 10^6^ cells/100 mm culture petri plate. At approximately 80–85% confluency, all culture medium was removed and the cells were washed 2× with PBS, then further incubated in culture medium (CM) (9.0 mL/plate) prepared in 5% Exo-Free FBS. Cells were incubated for an additional 48 h to allow the parent cells to release exosomes, at which point exo-free CM was collected for exosome isolation. 

#### 4.4.2. Preparation of Exo-Free FBS 

All serum/biological fluids contain exosomes. To avoid contamination from exogenous exosomes from fetal bovine serum (FBS), FBS was subjected to ultracentrifugation as described [46] to deplete exosomes from FBS (Exo-free FBS).

#### 4.4.3. Exosomes Isolation 

Exosomes isolated from HEK293T-WT cells were labeled as Exo-WT and exosomes isolated from HEK293T-CD19 CAR producer cells were labeled as Exo-CD19 CAR. Exosomes were isolated by ultracentrifugation method as described [46]. In brief, CM from cultured cells was subjected to centrifugation for 10 min at 300× *g*. Supernatant was collected and centrifuged for 10 min at 2000× *g*. Again, the supernatant was collected and centrifuged for 30 min at 10,000× *g*. Then the supernatant was ultra-centrifuged for 120 min at 100,000× *g*. Pellets were re-suspended in PBS and ultra-centrifuged again for 120 min at 100,000× *g*. Pellets containing exosomes were harvested and reconstituted in 250 µL PBS. Each centrifugation step was carried out at 4 °C. Exosomal protein content was measured by BCA (bicinchoninic acid) protein assay kit (Bio-Rad). We have previously confirmed that the nanoparticle fraction isolated by the above method is indeed exosomal by Western blot CD61 and CD81 markers and by Nano Track Analysis (NTA), which measures size as well as Brownian motion of particles [27].

#### 4.4.4. Exosomal Markers CD63 and CD81 Expression Analysis by Flow Cytometry

We used an exosomes analysis kit (ab239682, Abcam) and followed the recommended steps. Briefly, exosomes (10 µg) were taken into 50 µL PBS. Capture beads (100 µL) were mixed with 50.0 µL of exosomes in a cytometer tube. The mixture was incubated overnight at room temperature in the dark. Primary detection antibody CD81 (biotin-conjugated) (5.0 µL) was added and incubated at 2–8 °C for 1 h in the dark. Washing steps: 1.0 mL of assay buffer was added to samples, mixed by tapping, and centrifuged at 2000 rpm for 5.0 min. The supernatant was decanted and the sample pellet was reconstituted in 100 µL assay buffer. A secondary detection reagent (streptavidin-PE conjugated) was added at dilution 1:20 and incubated at 2–8 °C for 25–30 min in dark. After washing, the samples were reconstituted in 350 µL assay buffer and analyzed by flow cytometry [22]. 

#### 4.4.5. Expression of CD19 CAR Markers Proteins on Exosomes by Flow Cytometry 

CD63 antibody-coated beads were labeled with exosomes, and then expression of endogenous eGFP on exosomes was visualized without eGFP antibody staining, under FITC channel of the flow cytometer. Expression of CD3z on exosomes was observed by staining with CD3z antibody using CD63 antibody-coated beads (ab239682, Abcam). Exosomes (10 µg) were taken into 50 µL PBS and mixed with 100 µL of capture beads in a cytometer tube. The mixture was incubated at room temperature overnight in dark. Washing: 1.0 mL of assay buffer was added to samples, mixed by tapping, and centrifuged at 2500× *g* for 5.0 min. The supernatant was decanted and the mixture/pellet was reconstituted in 100 µL assay buffer. Staining: CD3z-PE-conjugated antibody was added at dilution 1:20 and incubated at 2–8 °C for 20–30 min in the dark and washing steps were repeated. Sample pellets were reconstituted in 350 µL assay buffer and analyzed by the flow cytometer. 

#### 4.4.6. Exosomal RNA Extraction 

Total RNA was extracted from the purified exosomes by Trizol method (Invitrogen) as we previously published [27]. RNA was estimated by NanoDrop ND1000 spectrophotometer. Subsequently, 1–5 µg of exosomal RNA was taken for cDNA synthesis utilizing Oligo-dT primers and M-MLV reverse transcriptase (28025-013, Invitrogen). 

### 4.5. Exo-CD19 CAR Induced Cytotoxicity (Contact-Dependent) in CD19-Positive B-Leukemia Cells

We next wanted to confirm that exposure of CD19 CAR exosomes with target cells are functional after entering CD19 expressing leukemic B-cells and investigated pro-apoptotic gene expression profiles in CAR exosome exposed cell lines.

#### 4.5.1. Gene Expression by qPCR

HL-60 and K-562 (CD19-negative control cell lines) and JM1, Sup-B15, REH, NALM-6 (CD19-positive leukemia cell lines) were seeded (0.2 × 10^6^ cells/well in 96-well culture plate). Exo-WT and Exo-CD19 CAR were added to the cells (2.5 µg/µL exosomes) in a final culture volume of 100 µL for co-cultured. CAR-exosome exposed cells were harvested at two time points (day 1 and day 2) for cellular RNA extraction and cDNA preparation. TaqMan qPCR method was employed to amplify pro-apoptotic genes (BAD, BAX, and Caspase-3) using cellular cDNA derived from exposed cell lines as a PCR template, and Eurogentec master mix. Primer sequences, probe numbers, and gene accession numbers from the universal probe library (UPL, Roche Applied Science) are listed (Table 2). Fold change was calculated by comparing control (Exo-WT) vs. treated (Exo-CD19 CAR). Data was analyzed with RQ manager version 1.2.1 (Applied Biosystems) and expressed as fold change. GAPDH was utilized as a house-keeping gene.

#### 4.5.2. Cytotoxicity Measurement by MTS Assay

Different cells (HL-60, K-562, JM1 and SUP-B15, REH, and NALM-6) were seeded in a 96-well culture plate (cell density: 0.1 million/100 μL). Cells were treated with Exo-WT and Exo-CD19 CAR (2.5 µg/µL) and placed in incubator. On day 4, cytotoxicity was measured by MTS assay (cat# G3582, CellTiter 96^®^ aqueous one solution, Promega). Three independent experiments were carried out in duplicates; cumulative data represented is of each value (Mean ± SD). 

### 4.6. Statistical Analysis 

To compare the mean values between the two groups, unpaired *t*-test was used. Statistical significance was defined as *p* < 0.05. All results are represented as Mean ± SD. Each experiment was performed independently 3–5 times to confirm the reproducibility.

## 5. Conclusions

Our novel exosome CAR constructs expressing CD19 CAR (Exo-CD19 CAR) can target and induce cell death in CD19-positive malignant B-cells without inducing cytotoxicity in CD19-negative cells. The current innovation uses nanovesicles called exosomes instead of whole CD19 CAR T-cell. The concept of customized made CAR-plasmid transfected exosomes is not limited to a cure for the hematological CD19 B-cell malignancies but can be possibly utilized for other CD19 B-cell expressing conditions such as in autoimmune diseases. 

## 6. Patents

Provisional patent has been filed by the Feinstein Institute for Medical Research, Northwell Health on 4 December 2020.

## Figures and Tables

**Table 1:** List of human primers for semi-quantitative PCR.

SN	Gene Name	Gene ID	Primers
1	eGFP	JQ064507.1	For 5′-CTGGTCGAGCTGGACGGCGACG-3′
			Rev 5′-CACGAACTCCAGCAGGACCATG-3′
2	CD8a	NM_001768.6	For 5′-CACGACGCCAGCGCCGCGACCACC-3′
			Rev 5′-GGGTGATAACCAGTGACAGGAGAA-3′
3	CD28	AJ937363.1	For 5′-GGAGGGGGGACCAAGCTGGAGA-3′
			Rev 5′-TGCAGACTGTTCATTTTTAAG-3′
4	β-actin	NM_001101.4	For 5′-GTCCTCTCCCAAGTCCACACA-3′
			Rev 5′-CTGGTCTCAAGTCAGTGTACAGGTAA-3′

**Table 2:** List of human primers for qPCR.

SN	Gene Name	Gene ID	Primers	UPL #
1	BAD	AF031523.1	For: 5′-CGAGTTTGTGGACTCCTTTAAGA-3′	78
			Rev: 5′-CACCAGGACTGGAAGACTCG-3′	
2	BAX	U19599.1	For: 5′-CAAGACCAGGGTGGTTGG-3′	55
			Rev: 5′-CACTCCCGCCACAAAGAT-3′	
2	Caspase-3	DD346274.1	For: 5′-AATGGACCAGGACGATGAAG-3′	35
			Rev: 5′-CATCTCATCACCCACTGCTC-3′	
4	GAPDH	NM_002046.3	For: 5′-AGCCACATCGCTCAGACAC-3′	60
			Rev: 5′-GCCCAATACGACCAAATCC-3′	
5	IFN-g	X13274.1	For: 5′-GGCATTTTGAAGAATTGGAAAG-3′	21
			Rev: 5′-TTTGGATGCTCTGGTCATCTT-3′	
6	TNF-a	X02910.1	For: 5′-GTCCAGGCTTGTCCTGCTAC-3′	7
			Rev: 5′-AGTCCTGAGGCCTGTGTTTG-3′	
7	IL-2	S77834.1	For: 5′-AAGTTTTACATGCCCAAGAAGG-3′	65
			Rev:5′-AAGTGAAAGTTTTTGCTTTGAGCTA-3′	

## Data Availability

Not applicable.

## Supplementary Materials

The following are available online at https://www.mdpi.com/2072-6694/13/6/1401/s1. Figure S1: Characterization and confirmation of CD19 CAR plasmid construct. Figure S2: Exosomes reached plateau at 2.5 μg/μL dose. Figure S3: Densitometry of agarose gels by Image J. Figure S4: Expression of cytokines (IFN-g, TNF-a, and IL-2) mRNA by q-PCR. Figure S5: Exo-CD19 CAR effect on CD19 negative and CD19 positive cells. Figure S6: Full image of Figure 2A. Figure S7: Full image of Figure 4A. Figure S8: Full image of Figure 5A (HL-60). Figure S9: Full image of Figure 5B (K-562). Figure S10: Full image of Figure 5C (JM1). Figure S11: Full image of Figure 5D (Sup-B15). Figure S12: Full image of Figure 5E (REH). Figure S13: Full image of Figure 5F (NALM-6).
