# Supplementary material for: CD19 Chimeric Antigen Receptor-Exosome Targets CD19 Positive B-lineage Acute Lymphocytic Leukemia and Induces Cytotoxicity
Source: Cancers (Basel). 2021 Mar 19;13(6):1401. doi: 10.3390/cancers13061401 (PMC8003442; doi:10.3390/cancers13061401)

CD19 Chimeric Antigen Receptor-Exosome Targets CD19 Positive B-lineage Acute Lymphocytic Leukemia and Induces Cytotoxicity

Shabirul Haque [1,2,*] and Sarah R. Vaiselbuh [1,2,3]

[1] Feinstein Institute for Medical Research, Northwell Health, 350 Community Drive, Manhasset, NY 11030, USA; svaiselbuh1@pride.hofstra.edu
[2] Department of Pediatrics, Staten Island University Hospital, Northwell Health, 475 Seaview Ave,
Staten Island, NY 10305, USA
[3] Current affiliation: Monsey Health Center, 40 Robert Pitt Drive, Monsey, NY 10952, USA
* Correspondence: shaque@northwell.edu

Supplementary materials

Supplemental Fig. S1

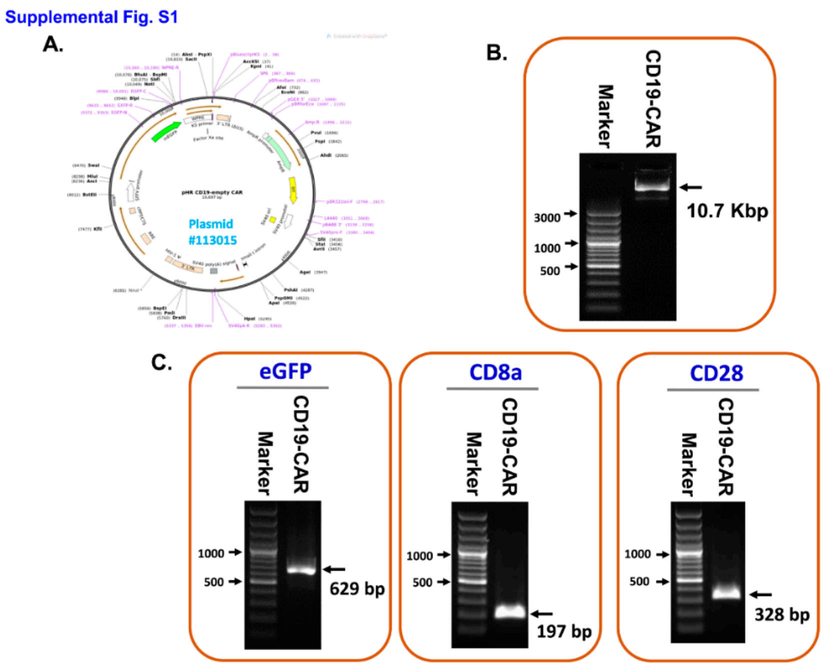

Supplemental Figure S1

Characterization and confirmation of CD19 CAR plasmid construct.

A. Schemata diagram of CD19 CAR plasmid construct.

B. Agarose gel showing electrophoretic pattern of intact plasmid DNA of CD19 CAR construct.

C. Agarose gel electrophoresis showing PCR amplified product of eGFP, CD8a, and CD28 using plasmid DNA as template.

Supplemental Fig. S2

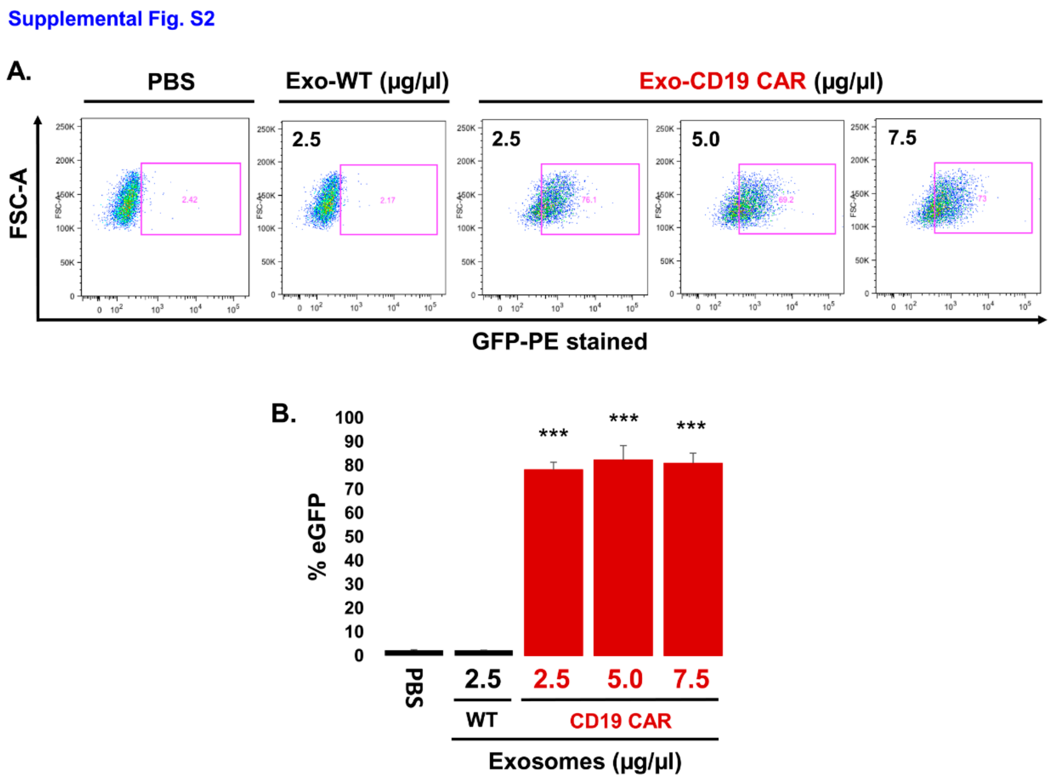

Supplemental Figure S2

Exosomes reached plateau at 2.5 µg/µl dose.

A. Sup-B15 cells were seeded (0.1x106 cells/well in 96 well culture plate). Three different doses, 2.5, 5.0, and 7.5 µg/µl of exosomes (Exo-WT and Exo-CD19 CAR) was added to the target cells and cocultured for 2 days. Cultured cells were harvested and stained with eGFP antibody. Expression of eGFP was analyzed by flow cytometry.

B. Bar graphs are representative of triplicates. P value (***p<0.001).

Supplemental Fig. S3

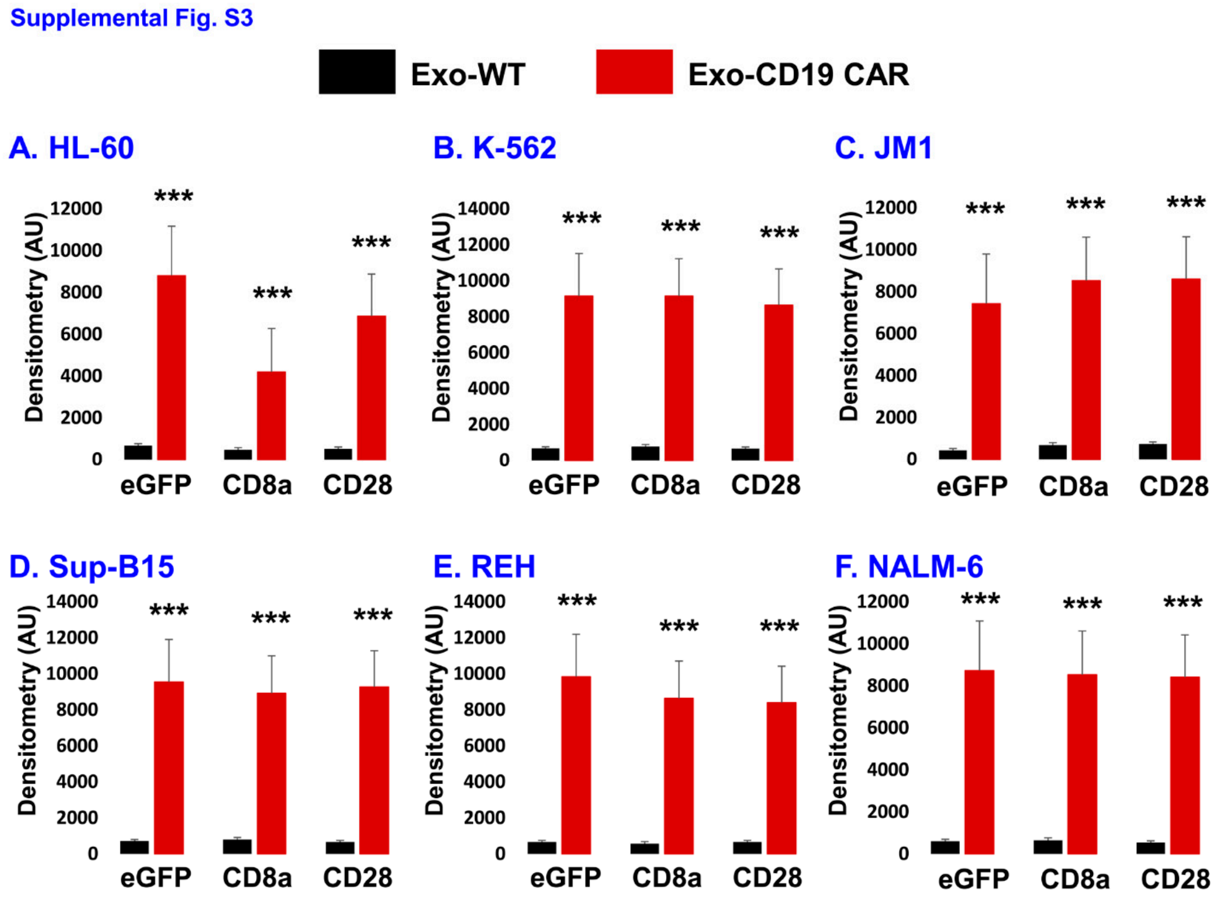

Supplemental Figure S3

Densitometry of agarose gels by Image J.

Densitometry of each gels of Figure 5 was carried out by Image J. Bar graphs are representative of five replicates. P value (***p<0.001).

Supplemental Fig. S4

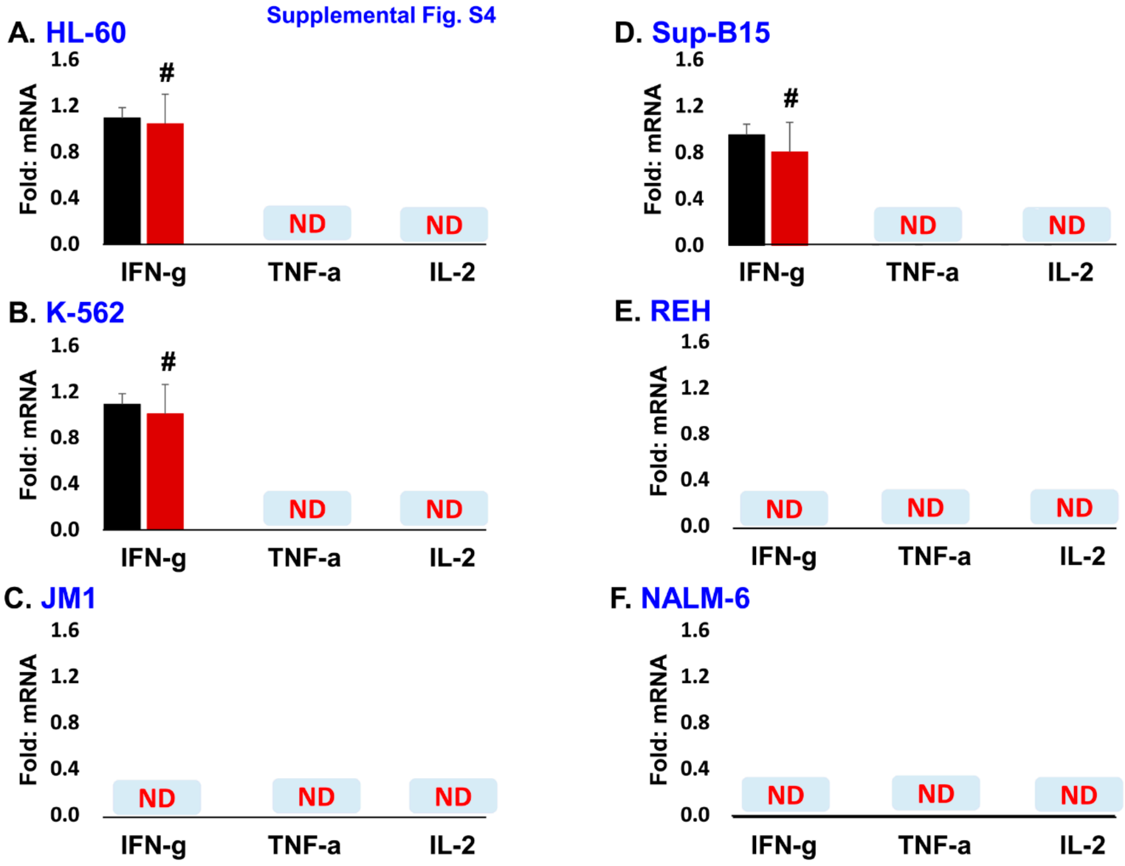

Supplemental Figure S4

Expression of cytokines (IFN-g, TNF-a, and IL-2) mRNA by q-PCR. Exo-CD19 CAR exposure does not induce cytokine genes compared to Exo-WT. **A-B**. Exo-CD19 CAR exposure could not induce cytokine genes (IFN-g, TNF-a, and IL-2) in CD19 negative cell lines (HL-60 and K-562). Further, **C-F.** Exo-CD19 CAR treatment could not induce cytokine genes (IFN-g, TNF-a, and IL-2) in CD19 positive cell lines (JM1, Sup-B15, REH, NALM-6). Representative data is pooled from three different experiments (n=3). Statistical p-value, # represents not significant. RED bars demonstrate Exo-CD19 CAR exposure, and BLACK bars demonstrate control Exo-WT treatment. ND denotes not detected/determined. The Ct values (32 - 35) were utilized for the calculation of IFN-g mRNA fold expression which shows very low level of expression. Ct value more than 35 considered as not determined.

Supplemental Fig. S5

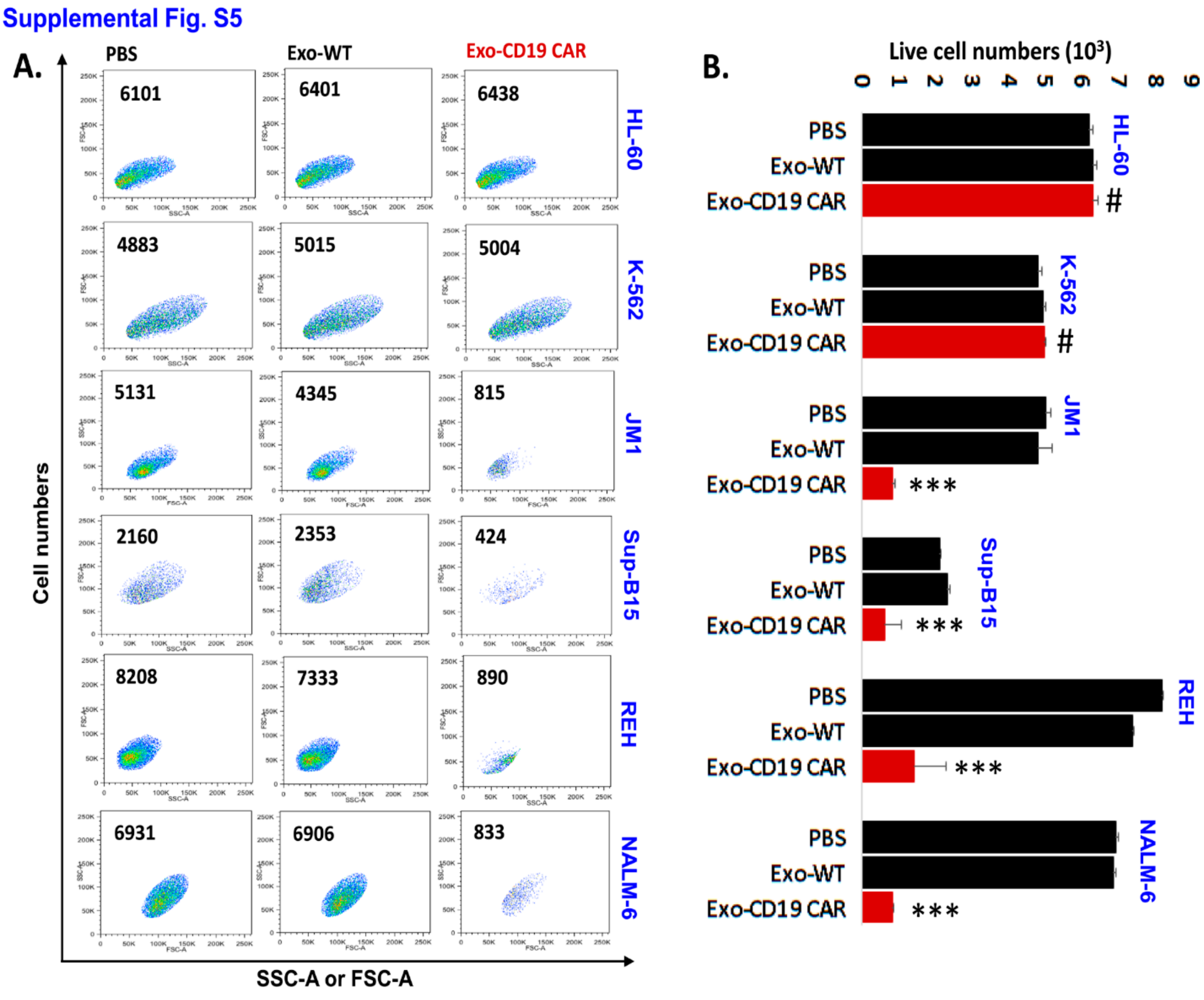

Supplemental Figure S5

Exo-CD19 CAR effect on CD19 negative and CD19 positive cells.

A. Exo-CD19 CAR exposure induces cytotoxicity in CD19 positive target cells (JM1, Sup-B15, REH, and NALM-6) while no cytotoxicity was seen in CD19 negative target cells (HL-60, and K-562) demonstrated by cell count on flow cytometry.

B. Computed data (n=3 exp) demonstrating level of significance and reproducibility. Exo-CD19 CAR could not induce cytotoxicity in CD19 negative cells while significant cytotoxicity was induced in CD19 positive cells. P value (***p<0.001). # represents not-significant (p>0.05).

Supplemental Fig. S6

Full image of Fig 2A

Supplemental Fig. S6

Fig 2 A Full image of electrophoresed PCR products on agarose (1.5%) gel

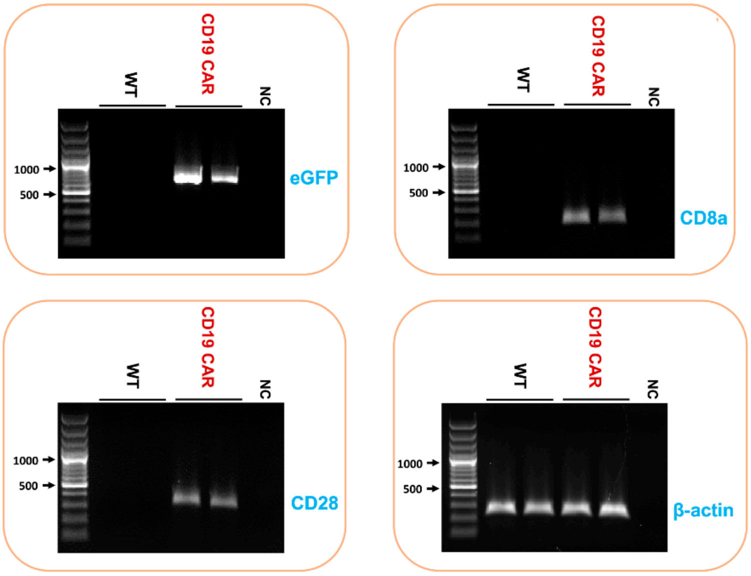

Supplemental Fig. S7

Full image of Fig 4A

Supplemental Fig. S7

Fig 4 A Full image of electrophoresed PCR products on agarose (1.5%) gel

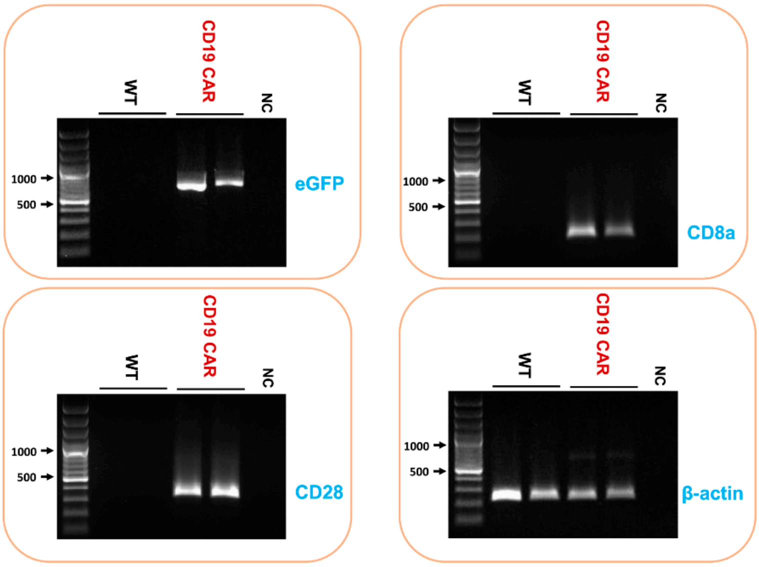

Supplemental Fig. S8

Full image of Fig 5A (HL-60)

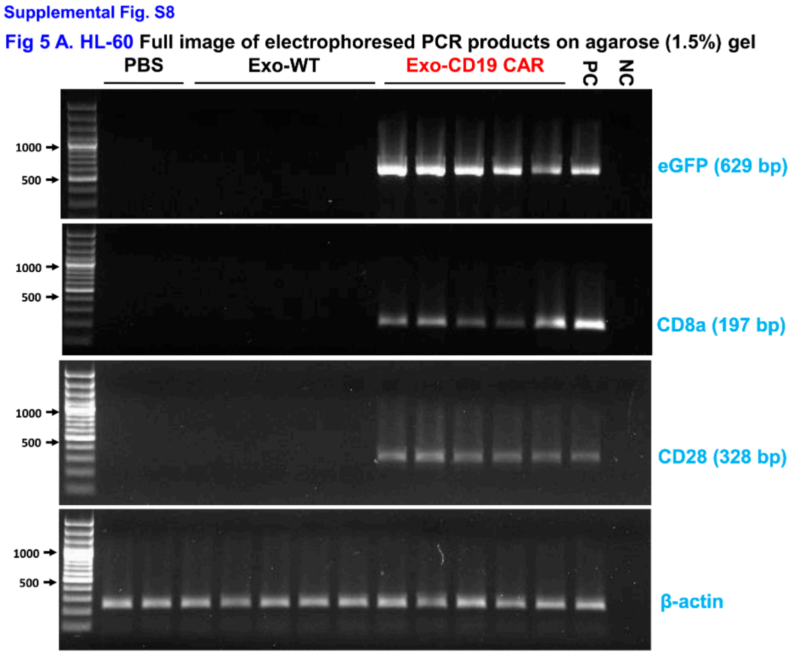

Supplemental Fig. S9

Full image of Fig 5B (K-562)

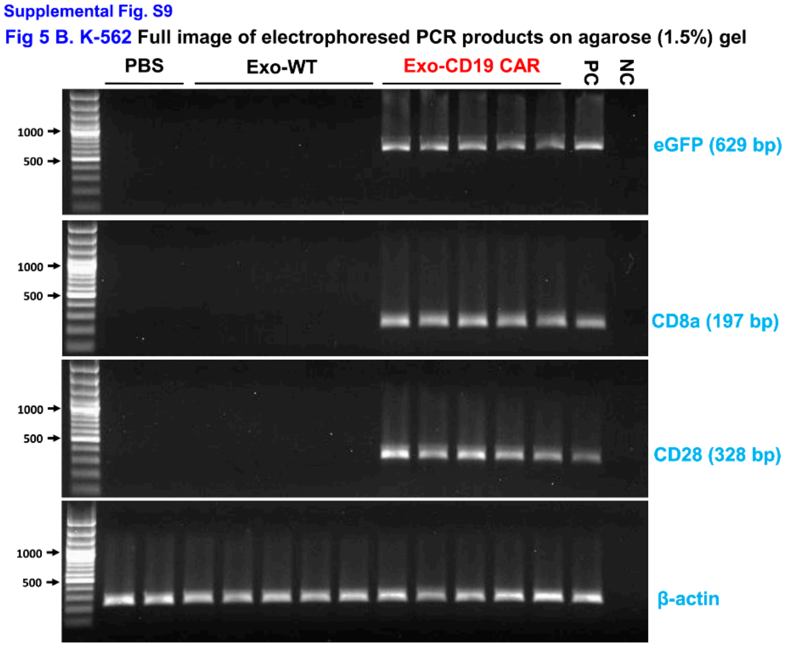

Supplemental Fig. S10

Full image of Fig 5C (JM1)

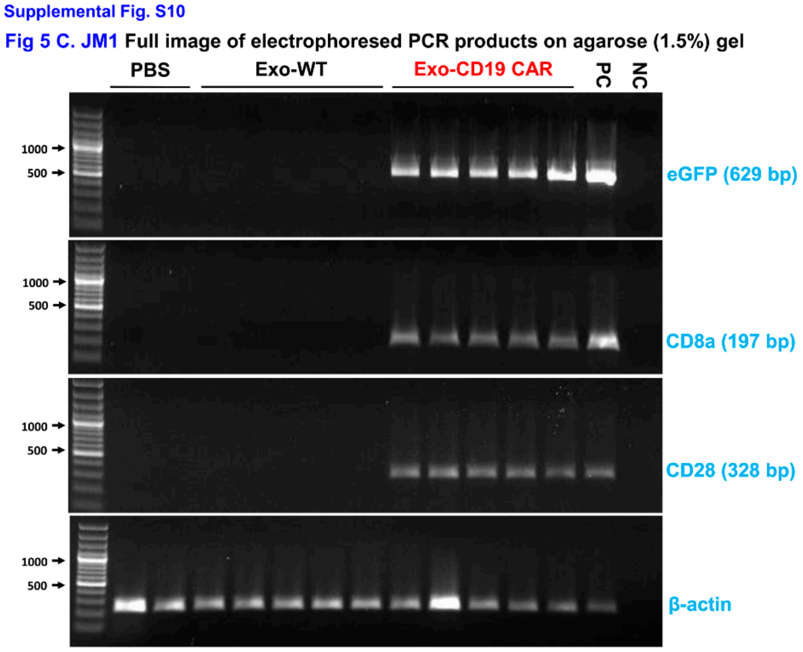

Supplemental Fig. S11

Full image of Fig 5D (Sup-B15)

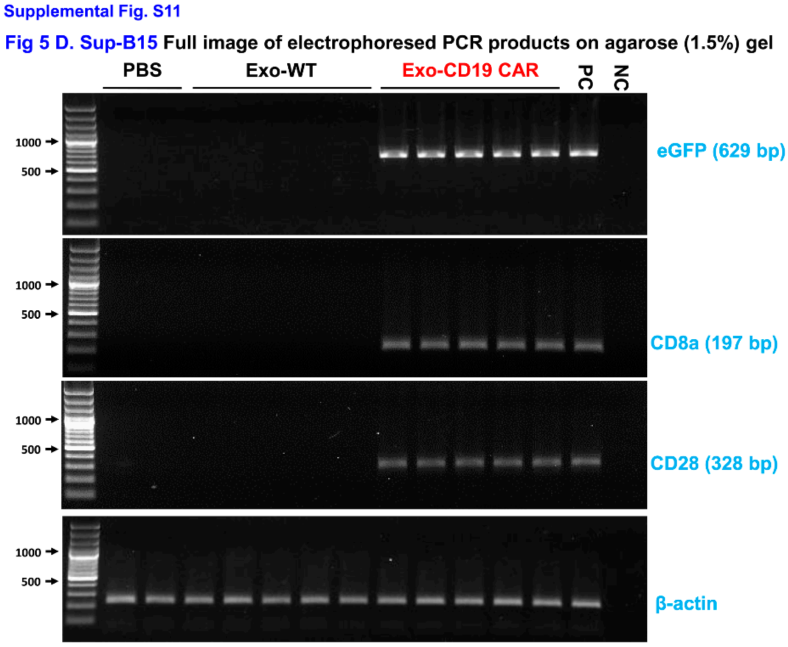

Supplemental Fig. S12

Full image of Fig 5E (REH)

Supplemental Fig. S12

Fig 5 E. REH Full image of electrophoresed PCR products on agarose (1.5%) gel

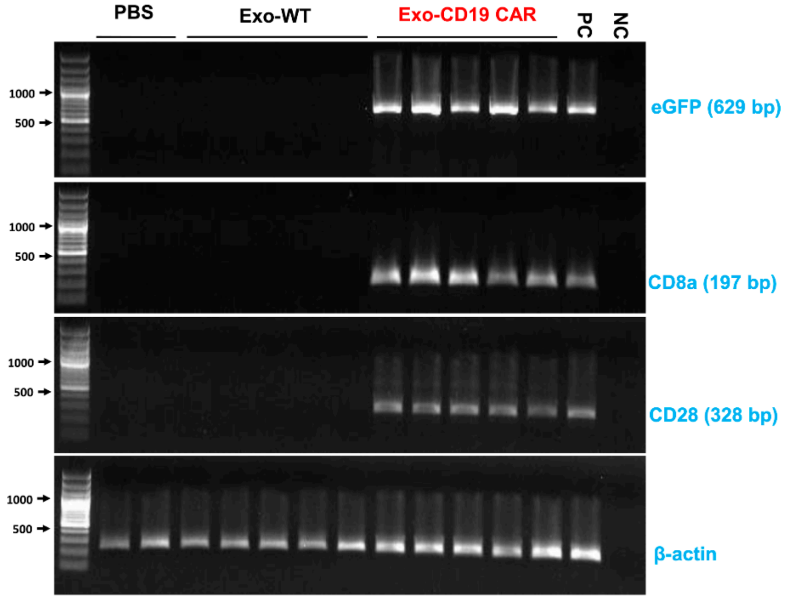

Supplemental Fig. S13

Full image of Fig 5F (NALM-6)

Supplemental Fig. S13

Fig 5 F. NALM-6 Full image of electrophoresed PCR products on agarose (1.5%) gel

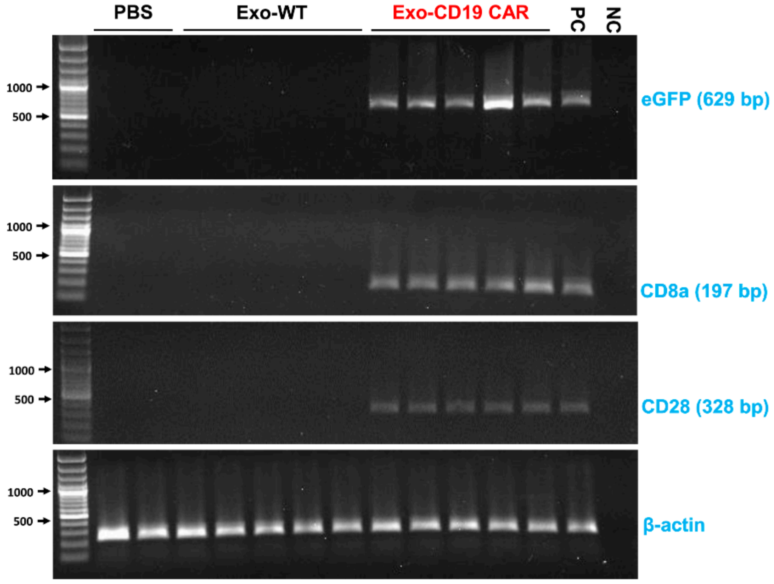

Supplement: Supplementary file 1 [file cancers-13-01401-s001.pdf]
